# Complete mitochondrial genome sequence of the green alga *Mychonastes koreanus* MKSC25 (Chlorophyta)

**DOI:** 10.1128/mra.00838-26

**Published:** 2026-10-27

**Authors:** Nam Seon Kang, Chang Rak Jo, Sun Young Kim, Biet Thanh Tran, Taeho Lee, Jung Soo Heo, Jinwook Back, Keun-Yong Kim, Sung Min An

**Affiliations:** 1 National Marine Biodiversity Institute of Korea, Seocheon, Republic of Korea; 2 Bioinformatics Team, AquaGenTech Co., Ltd., Busan, Republic of Korea; 3 Department of Civil and Environmental Engineering, Pusan National University, Busan, Republic of Korea; University of Strathclyde, Glasgow, United Kingdom

**Keywords:** mitogenome, phylogeny, green algae, Chlorophyceae, *Mychonastes koreanus*

## Abstract

The complete mitochondrial genome of the green alga *Mychonastes koreanus* MKSC25 is 23,905 bp long, with a GC content of 44.88%. It contains 13 protein-coding genes, 22 tRNA genes, 2 rRNA genes fragmented into 7 segments, 1 putative open reading frame (*orf158*), and a 299-bp group I intron interrupting *rrnL4*.

## ANNOUNCEMENT

Members of the genus *Mychonastes* (Chlorophyta; Chlorophyceae) inhabit freshwater and brackish habitats (1, 2). Although *Mychonastes koreanus* was originally described from freshwater (3), strain MKSC25 was isolated from coastal seawater, suggesting tolerance to saline conditions. Here, we report the mitochondrial genome sequence of *M. koreanus* MKSC25 as a resource for comparative mitogenomic and phylogenetic studies within *Mychonastes*.

*M. koreanus* MKSC25 was isolated from coastal seawater in Seocheon-gun, Republic of Korea (36°01′46.55″N, 126°39′51.14″E) in June 2025 (25°C, 32 PSU). A unialgal culture was established by repeated streaking on BG-11 agar (1.5%) (2). For genomic DNA extraction, strain MKSC25 was cultured in 100 mL BG-11 medium at 27°C for 21 days under a 14-h light/10-h dark photoperiod with an irradiance of 170 μmol photons m⁻² s⁻¹. Genomic DNA was extracted using the APBIO APrep Plant DNA Kit (APBIO, Namyangju-si, Republic of Korea).

The strain was identified based on morphology (2) and sequences of the 18S rRNA gene, ITS region, and 28S rRNA gene (GenBank accession numbers PZ195265, PZ195266, and PZ195267), showing 100% sequence identity to the original species description (3). A specimen was deposited at the National Marine Biodiversity Institute of Korea, Seocheon (https://www.mabik.re.kr/) under voucher number MABIK LP00000468.

A paired-end library was prepared with the Illumina DNA Prep kit and sequenced (2 × 150 bp) on a NovaSeq 6000 System (Illumina, San Diego, CA, USA) using the NovaSeq 6000 S4 Reagent Kit v1.5. Raw reads (316,197,660 reads; 47.75 Gb) were processed using Cutadapt v3.11 (4) to trim adapters and filter low-quality sequences (*Q* < 20). The cleaned reads (280,424,160 reads) were *de novo* assembled in CLC Genomics Workbench v2025 (CLC bio, Aarhus, Denmark) using a similarity fraction of 0.8 and a length fraction of 0.5, generating 1,654 contigs (23,784,083 bp; N50, 28,763 bp). Contigs showing BLASTN similarity (*E*-value ≤0.001) to published chlorophyte mitochondrial genes were identified by the BLASTN algorithm (5) implemented in CLC Genomics Workbench. The cleaned reads were iteratively mapped to the mitochondrial contigs in Geneious Prime v2021.1.1 (Biomatters, Auckland, New Zealand) using the default mapper settings until no further extension was achieved. Circularization was confirmed by manual inspection of overlapping terminal sequences, after which the redundant overlap was trimmed and the genome was rotated to begin with the *co1* gene. Genes were annotated using GeSeq v2.03 (6) and manually verified in MEGA v10.2.2 (7). Sequencing depth and coverage were assessed using Qualimap v2.3 (8), and the mitogenome map was generated using OGDRAW v1.3.1 (9).

**Fig 1:**
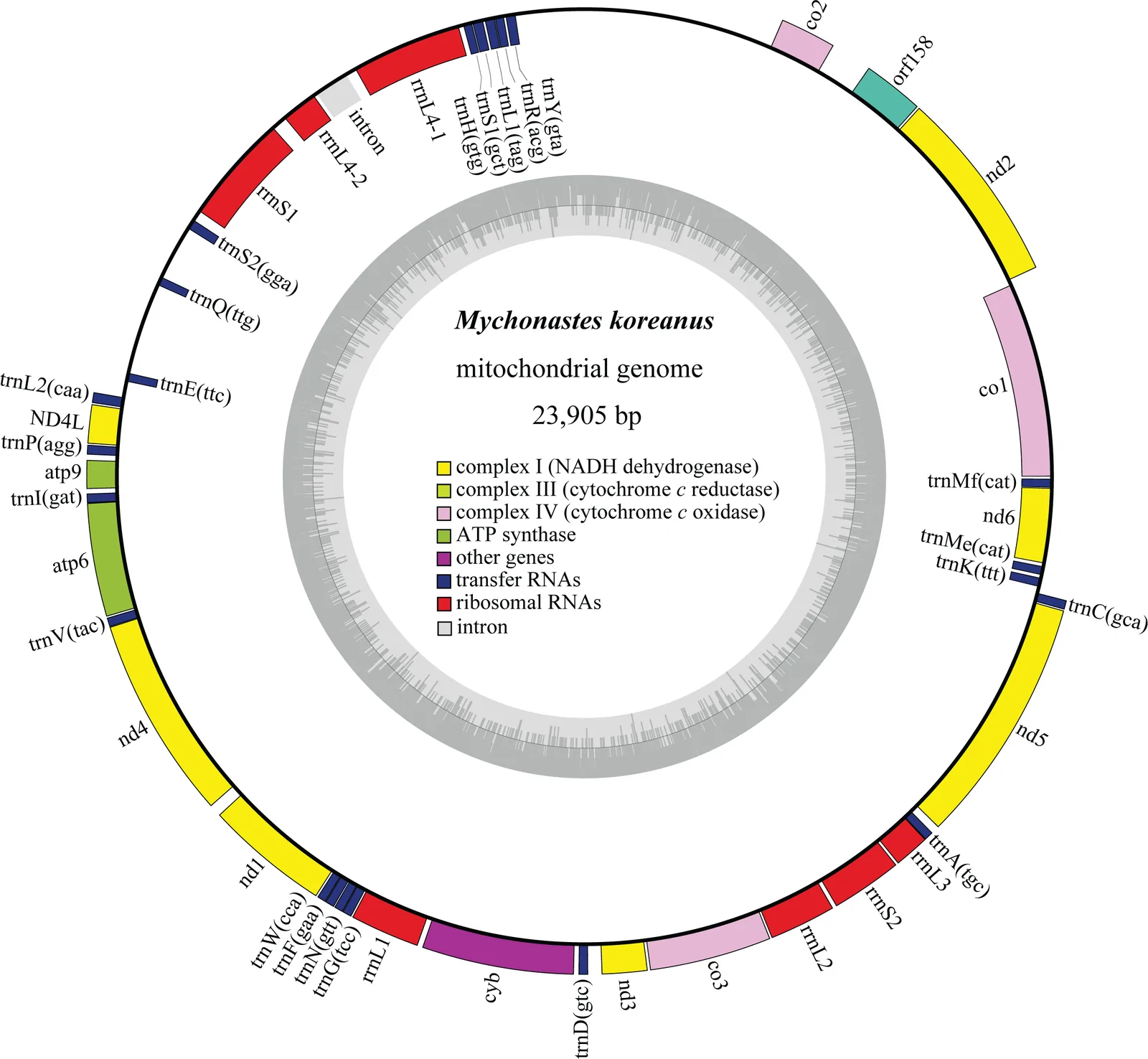
Circular mitochondrial genome of *Mychonastes koreanus* MKSC25 (GenBank accession number PZ368384). Genes encoded on the reverse and forward strands are illustrated inside the circle and outside the circle, respectively. The inner gray track displays the GC content across the genome.

The complete mitogenome of *M. koreanus* MKSC25 (Fig. 1) is a circular double-stranded DNA molecule of 23,905 bp with a GC content of 44.88%. The assembly achieved 100% coverage at an average depth of 2,880×. The genome contains 13 protein-coding genes, 22 tRNA genes, 2 rRNA genes fragmented into 7 segments, one 477-bp putative open reading frame (*orf158*), and a 299-bp group I intron interrupting *rrnL4* (Table 1). Among the 13 protein-coding genes, 12 terminate with TCA, whereas only *co3* terminates with TAA, consistent with related chlorophycean mitochondrial genomes (10).

**TABLE 1:** Annotation summary and gene organization of the *Mychonastes koreanus* MKSC25 mitochondrial genome^*a*^

Gene	Type	Start position	End position	Length	Direction	Start/Stop codon
*co1*	CDS	1	1608	1608	Reverse	ATG/TCA
*nd2*	CDS	1650	3173	1524	Forward	ATG/TCA
*orf158*	Putative ORF	3188	3664	477	Forward	ATG/TGA
*co2*	CDS	3983	4420	438	Forward	ATG/TCA
*trnY(gta)*	tRNA	6540	6621	82	Reverse	–
*trnR(acg)*	tRNA	6637	6710	74	Reverse	–
*trnL1(tag)*	tRNA	6713	6794	82	Reverse	–
*trnS1(gct)*	tRNA	6810	6897	88	Reverse	–
*trnH(gtg)*	tRNA	6902	6974	73	Reverse	–
*rrnL4-1*	rRNA fragment	7013	8004	992	Reverse	–
intron	intron	8005	8303	299	Reverse	–
*rrnL4-2*	rRNA fragment	8304	8325	22	Reverse	–
*rrnS1*	rRNA fragment	8747	9656	910	Reverse	–
*trnS2(gga)*	tRNA	9741	9824	84	Reverse	–
*trnQ(ttg)*	tRNA	10274	10346	73	Reverse	–
*trnE(ttc)*	tRNA	11101	11173	73	Reverse	–
*trnL2(caa)*	tRNA	11304	11386	83	Forward	–
*nd4l*	CDS	11397	11699	303	Forward	ATG/TCA
*trnP(agg)*	tRNA	11709	11783	75	Forward	–
*atp9*	CDS	11825	12046	222	Forward	ATG/TCA
*trnI(gat)*	tRNA	12082	12154	73	Forward	–
*atp6*	CDS	12155	13036	882	Forward	ATG/TCA
*trnV(tac)*	tRNA	13047	13118	72	Forward	–
*nd4*	CDS	13120	14703	1584	Forward	ATG/TCA
*nd1*	CDS	14795	15763	969	Forward	ATG/TCA
*trnW(cca)*	tRNA	15776	15846	71	Forward	–
*trnF(gaa)*	tRNA	15848	15921	74	Forward	–
*trnN(gtt)*	tRNA	15927	15998	72	Forward	–
*trnG(tcc)*	tRNA	16000	16073	74	Forward	–
*rrnL1*	rRNA fragment	16084	16627	544	Forward	–
*cyb*	CDS	16667	17848	1182	Forward	ATG/TCA
*trnD(gtc)*	tRNA	17884	17957	74	Forward	–
*nd3*	CDS	18061	18414	354	Forward	ATG/TCA
*co3*	CDS	18433	19383	951	Forward	ATG/TAA
*rrnL2*	rRNA fragment	19397	19917	521	Forward	–
*rrnS2*	rRNA fragment	19947	20503	557	Forward	–
*rrnL3*	rRNA fragment	20521	20799	279	Forward	–
*trnA(tgc)*	tRNA	20800	20872	73	Forward	–
*nd5*	CDS	20934	22868	1935	Forward	ATG/TCA
*trnC(gca)*	tRNA	22877	22949	73	Reverse	–
*trnK(ttt)*	tRNA	23001	23074	74	Reverse	–
*trnMe(cat)*	tRNA	23099	23170	72	Reverse	–
*nd6*	CDS	23196	23813	618	Reverse	ATG/TCA
*trnMf(cat)*	tRNA	23817	23888	72	Reverse	–

^*a*^ –, not applicable.

## Data Availability

The mitogenome sequence generated in this study is available in the GenBank database of NCBI (accession number PZ368384). The associated BioProject, SRA, and BioSample numbers are PRJNA1454689, SRR38145619, and SAMN57317157, respectively.
